# Laparoscopic right hemicolectomy: the SICE (Società Italiana di Chirurgia Endoscopica e Nuove Tecnologie) network prospective trial on 1225 cases comparing intra corporeal versus extra corporeal ileo-colic side-to-side anastomosis

**DOI:** 10.1007/s00464-019-07255-2

**Published:** 2019-11-18

**Authors:** Gabriele Anania, Ferdinando Agresta, Elena Artioli, Serena Rubino, Giuseppe Resta, Nereo Vettoretto, Wanda Luisa Petz, Carlo Bergamini, Alberto Arezzo, Giorgia Valpiani, Chiara Morotti, Gianfranco Silecchia, SICE CoDIG (Colon Dx Italian Group)

**Affiliations:** 1 Division of General Surgery, S. Anna University Hospital of Ferrara, via Aldo Moro 8, Cona, FE Italy; 2 Department of Morphology, Surgery and Experimental Medicine, University of Ferrara, Ferrara, Italy; 3 Department of General Surgery, ULSS5 Polesana del Veneto, Adria, RO Italy; 4 Montichiari Surgery, ASST Spedali Civili Brescia, Montichiari, BS Italy; 5 Division of Gastrointestinal Surgery, IEO, European Institute of Oncology IRCCS, Milan, Italy; 6 Department of Emergency Surgery, University Hospital of Careggi, Florence, Italy; 7 Department of Surgical Sciences, University of Torino, Turin, Italy; 8 MsC in Statistics at Research Innovation Office, S. Anna University Hospital of Ferrara, Ferrara, Italy; 9 Department of Medical Surgical Science and Biotechnologies, Faculty Pharmacy and Medicine, Sapienza University of Rome, Rome, Italy

**Keywords:** Right hemicolectomy, Ileo-colic anastomosis, Laparoscopy, Postoperative complications, Intracorporeal anastomosis, Outcomes

## Abstract

**Background:**

While laparoscopic approach for right hemicolectomy (LRH) is considered appropriate for the surgical treatment of both malignant and benign diseases of right colon, there is still debate about how to perform the ileo-colic anastomosis. The ColonDxItalianGroup (CoDIG) was designed as a cohort, observational, prospective, multi-center national study with the aims of evaluating the surgeons’ attitude regarding the intracorporeal (ICA) or extra-corporeal (ECA) anastomotic technique and the related surgical outcomes.

**Methods:**

One hundred and twenty-five Surgical Units experienced in colorectal and advanced laparoscopic surgery were invited and 85 of them joined the study. Each center was asked not to change its surgical habits. Data about demographic characteristics, surgical technique and postoperative outcomes were collected through the official SICE website database. One thousand two hundred and twenty-five patients were enrolled between March 2018 and September 2018.

**Results:**

ICA was performed in 70.4% of cases, ECA in 29.6%. Isoperistaltic anastomosis was completed in 85.6%, stapled in 87.9%. Hand-sewn enterotomy closure was adopted in 86%. Postoperative complications were reported in 35.4% for ICA and 50.7% for ECA; no significant difference was found according to patients’ characteristics and technologies used. Median hospital stay was significantly shorter for ICA (7.3 vs. 9 POD). Postoperative pain in patients not prescribed opioids was significantly lower in ICA group.

**Conclusions:**

In our survey, a side-to-side isoperistaltic stapled ICA with hand-sewn enterotomy closure is the most frequently adopted technique to perform ileo-colic anastomosis after any indications for elective LRH. According to literature, our study confirmed better short-term outcomes for ICA, with reduction of hospital stay and postoperative pain.

**Trial registration:**

Clinical trial (Identifier: NCT03934151).

While laparoscopic approach for right hemicolectomy (LRH) is considered appropriate for the surgical treatment of both malignant and benign diseases of the right colon, there is still debate about how to perform the ileo-colic anastomosis [1–4].

Over time, different types of ileo-colic anastomosis, such as stapled, hand-sewn and hybrid techniques, have been described and compared. Side-to-side ileo-colic anastomosis is the most frequently used technique by open surgery as well as by minimally invasive approach [5].

Since the intra-corporeal ileo-colic anastomosis (ICA) technique has been proposed [6], the attention was focused on the comparison with the extra-corporeal option. Several cons of the extra-corporeal technique (ECA) have been advocated, such as prolonged paralytic ileus, longer postoperative stay, higher risk of wound infection and incisional hernia.

On the other hand, better short-term outcomes related to lower bowel manipulation and stretching, mini Pfannestiel incision used for specimen extraction, have been reported as advantages of the ICA [7–9]. However, ICA is associated to important technical issues: a longer learning curve and operative time as well as higher risk of peritoneal contamination [10].

So far, the only clinical evidence is offered by retrospective low powered studies that indicate faster bowel movements, earlier re-feeding and shorter postoperative hospital stay after LRH with ICA. Prospective comparative multi-center studies comparing perioperative outcomes of ECA versus ICA are still missing [11–27].

The main purpose of this prospective multi-center national study was to evaluate the actual preference of the surgeons concerning intracorporeal and extra-corporeal ileo-colic side-to-side anastomosis after LRH as well as to assess the perioperative complications related to the techniques.

## Materials and methods

### Study design

The CoDIG (ColonDxItalianGroup – Italian Right Colon Group) study was designed as a cohort, observational, prospective, multi-center national study comparing ileo-colic side-to-side ECA and ICA techniques after LRH. Patients were recruited from March 2018 to September 2018.

The study was approved and endorsed by SICE (Società Italiana di Chirurgia Endoscopica e Nuove Tecnologie – Italian Society of Endoscopic Surgery and New Technologies).

The Coordinator Center and Promoter of the study is the 1st Department of General Surgery of Ferrara University Hospital.

One hundred and twenty-five Surgical Units, with experience in colorectal laparoscopic surgery were invited and 85 of them (68%) joined the study. Data were collected using the official SICE website database.

Each center was asked not to change the current practice: the technologies used, the surgical approach, the anastomotic method, the pre- and postoperative management (ERAS protocol and opioid administration included). Patients involved into the study signed an informed consent.

The primary endpoint of the study was to assess the preference of the Italian surgeon (ECA vs. ICA) when performing the ileo-colic side-to-side anastomosis after any indication for LRH.

The secondary endpoint was to compare the postoperative hospital stay, time for re-feeding, time before bowel movement and rate of complications at 30 days after ECA or ICA in LRH.

### Population, inclusion/exclusion criteria, data extraction

Each center was committed to enroll all consecutive cases observed during the study period according to inclusion and exclusion criteria.

Inclusion criteria were age > 18 years old, elective laparoscopic/robotic right hemicolectomy.

Exclusion criteria were emergency surgery and Body Mass Index (BMI) > 35.

A section of the official SICE website allowed the online collection of the following data for each patient enrolled:

- Patient’s characteristics: gender, age, BMI, major co-morbidities (diabetes mellitus, ischemic cardiomyopathy, chronic obstructive arteriopathy, chronic pneumopathy)
- Previous abdominal surgery;
- Indication for surgery: benign or malignant diseases (site of the carcinoma, staging, number of lymph nodes retrieved, number of metastatic lymph nodes)
- Surgical technique: laparoscopy/robotic and the imaging technologies (Full HD, 3D, Indocyanine Green, energy devices) used;
- Type of anastomosis: ECA or ICA, stapled or hand-sewn (single or double layer), isoperistaltic or anti-peristaltic, stapled or hand-sewn enterotomies closure (interrupted or continuous suture);
- Intra-operative complications: hemorrhage, bowel iatrogenic lesions; conversion to open surgery
- Postoperative complications: hemorrhage, anastomotic leakage (method of assessment);
- Postoperative management: ERAS protocol, opioid administration
- Postoperative hospital stay, time for re-feeding, time to return of bowel functions, postoperative pain monitored at scheduled interval (6, 12, 24, 48 h) through Visual Analog Scale for Pain;
- Hospital readmission within 30 days for surgery related complications.

### Statistical analysis

Categorical data were expressed as total numbers and percentages. Statistical comparisons of categorical variables were assessed using Pearson’s *χ*^2^ test or Fisher’s exact test depending on the minimal expected count in each crosstab. Length of stay was represented with the median and interquartile range [1Q–3Q] and the Mann–Whitney test was used to analyze the difference between ICA and ECA groups. Unadjusted and adjusted logistic regression were used to estimate odds ratios and respective 95% confidence intervals (95% CIs). In the multivariate analysis we considered as dependent variable presence of postoperative complications and as covariate: gender, age, presence of previous abdominal surgery, comorbidity, ASA score, operative time and blood loss. Model calibration for multivariate analysis was assessed using the Hosmer–Lemeshow goodness-of-fit test. All analyses were performed using Stata 14.1 SE (Stata Corporation, College Station, Texas, USA). A two-sided *p* value < 0.05 was defined as statistically significant.

## Results

One thousand two hundred twenty-five patients were enrolled; 631 (51.5%) were males and 594 (48.5%) females. Among all patients, 26.6% were under 65 years old, and 41% were older than 75 years. Overweight patients were 37.3% of all cases, while 13.2% had a class I obesity.

The indication for elective right hemicolectomy was a benign disease in the 13% whereas in the residual 87% the diagnosis was colonic adenocarcinoma, located at the cecum in 39.8%, ascending colon in 37.6%, and transverse colon in 22.7%.

One major comorbidity was present in 22% of the sample, while 20% had at least 2 co-morbidities at the time of surgery. Previous abdominal surgery had been performed in 46.7% of the cases.

The distribution according to ASA score (American Society of Anaesthesiologists) was: 7.6% ASA I, 49.6% ASA II, 39.8% ASA III, 3% ASA IV (Table 1).

**Table 1:** Characteristics of patients

Type	Complications		*n* (%)
	Yes (489)	No (736)	
Gender			
Male	252 (51.5%)	379 (51.5%)	631 (51.5)
Female	237 (48.5%)	357 (48.5%)	594 (48.5)
Age categories			
< 65 years old	132 (27%)	194 (26.4%)	326 (26.6)
66–75 years old	152 (31.1%)	240 (32.6%)	392 (32.0)
> 76 years old	205 (41.9%)	302 (41%)	507 (41.4)
BMI			
< 30	421 (86.1%)	642 (87.2%)	1063 (86.8)
≥ 30	68 (13.9%)	94 (12.3%)	162 (13.2)
Co-morbidities^a^			
None	284 (58.1%)	426 (57.9%)	710 (58.0)
1	119 (24.3%)	153 (20.8%)	272 (22.2)
≥ 2	86 (17.6%)	157 (21.3%)	243 (19.8)
ASA score			
1	43 (8.8%)	50 (6.8%)	93 (7.6)
2	236 (48.2%)	371 (50.4%)	607 (49.6)
3	196 (40.1%)	292 (39.7%)	488 (39.8)
4	14 (2.9%)	23 (3.1%)	37 (3)
Previous abdominal surgery			
None	274 (56%)	379 (51.5%)	653 (53.3)
1	215 (44%)	357 (48.5%)	572 (46.7)
Pathology			
Benign	58 (11.9%)	99 (13.4%)	157 (12.8)
Malignant	431 (88.1%)	637 (86.6%)	1068 (87.2)
Location of the disease			
Cecum	207 (42.3%)	280 (38%)	487 (39.8)
Ascending colon	183 (37.4%)	277 (37.7%)	460 (37.6)
Trasverse colon	99 (20.3%)	179 (24.3%)	278 (22.7)
Total			1225 (100)

^a^Diabetes mellitus, ischemic cardiomyopathy, chronic obstructive arteriopathy, chronic pneumopathy

### Techniques and technologies

Surgical procedures were performed in the 92.3% by laparoscopy, while robotic technique was used in 7.7% of all cases. Conversion was required in 66 patients (5.4%), 6 of them performed with robotic technique. Four cases were converted because of intra-operative hemorrhage, 35 cases because of technical difficulties due to overweight, 26 due to massive adhesions and 1 for a iatrogenic intestinal lesion.

In 69.4% of the procedures these were carried out using a Full HD vision technology, in 25.1% using 3D vision technology, and in the residual 5.5% using 4 K vision technology. Indocyanine Green fluoroangiography was used in 10.4% of the procedures.

Monopolar/bipolar energy was employed in 5% of the procedures, radiofrequency energy devices in 43.7% and ultrasound energy devices in the remaining 51.3%.

Surgical procedures lasted up to 180 min in 61.2% of all cases, 180 to 270 min in 32.7%, and more than 270 min in 6.1%. Moreover the duration of surgical procedure is not influenced by the ICA or ECA technique (p value 0.467).

Intra-operative blood loss was < 200 ml in 94.3%, among 201 and 299 ml in 0.4% and > than 300 ml in 5.3 of all cases. Blood transfusions during surgery were administered in 6.4% of the procedures (Table 2).

**Table 2:** Descriptive analysis of technologies used and intervention variables

Variables	Type	*n* (%)	
Technique used	Laparoscopic	1131 (92.3%)	
	Robotic	94 (7.7%)	
Visual technology	Full HD	850 (69.4%)	
	3D	308 (25.1%)	
	4K	67 (5.5%)	
Indocyanin green	Yes	127 (10.4%)	
	No	1098 (89.6%)	
Energy devices	Radiofrequency	536 (43.7%)	
	Ultrasound	628 (51.3%)	
	Monopolar/bipolar	61 (5%)	
Length of intervention	Minutes	ICA (862)	ECA (363)
	90–180	519 (60.2%)	231 (63.6%)
	180–270	287 (33.3%)	113 (31.1%)
	> 270	56 (6.5%)	19 (5.2%)
Blood loss (ml)	0–200	1155 (94.3%)	
	201–299	5 (0.4%)	
	≥ 300	65 (5.3%)	
Lymph nodes removed	< 12	79 (6.4%)	
	≥ 12	981 (80.1%)	
	Missing	165 (13.5%)	

### Anastomosis

ICA was performed in 862 cases (70.4%), ECA in 363 cases (29.6%).

In ICA group, isoperistaltic anastomosis was completed in 88.4% of patients, stapled in 97%. Concerning enterotomies closure, a manual suture was adopted in 95.7% of the cases (79% double layer, 17.2% single layer, with continuous suture in 88.4%).

In ECA group, isoperistaltic anastomosis was performed in 78.8%, stapled in 66.4%. Manual enterotomy closure was adopted in 62.8% (50.7% double layer, 17.1% single layer, with continuous suture in 50.4%) (Table 3).

**Table 3:** Characteristic of anastomosis

Variables	Type	ICA (%)	ECA (%)
Direction	Anisoperistaltic	100 (11.6)	77 (21.2)
	Isoperistaltic	762 (88.4)	286 (78.8)
Side-to-side	Manual	26 (3)	122 (33.6)
	Mechanical	836 (97)	241 (66.4)
Enterotomy closure	Manual	825 (95.7)	228 (62.8)
	Mechanical	37 (4.3)	135 (37.2)
Enterotomy manual closure	Single layer	148 (17.2)	62 (17.1)
	Double layer	681 (79)	184 (50.7)
	missing	33 (3.8)	117 (32.2)
	Continuous suture	762 (88.4)	183 (50.4)
	Interrupted suture	36 (4.2)	58 (16)
	Missing	64 (7.4)	122 (33.6)
Mesocolon closure	No	378 (43.8)	140 (38.6)
	Yes	484 (56.2)	223 (61.4)

### Intra-operative complications

Intra-operative complications were reported in 20 (1.6%) patients; 4 of which (1.3%) were intraperitoneal hemorrhages and 1 (0.3%) iatrogenic small bowel lesion.

### Postoperative outcomes

ERAS protocol was applied to 655 patients (53.5%), of which 537 belonging to ICA group (62.3% of ICA group) and 118 belonging to ECA group (32.5% of ECA group).

In 22.8% of patients gas passed during the first postoperative day (POD), in 66.4% between the second and the third POD, in 10.8% after the fourth POD.

A liquid diet was administered during the first POD to 11.1% of the patients, to 61.4% between the first and the second POD, to 19.4% between third and fourth POD and to 8.1% after the fourth POD.

A solid diet was restored on the same day of the operation in 0.8% of patients, between the first and the second POD in 37.6%, between the third and the fourth POD in 41.1% and after the fourth POD in 20.6%.

Patients were discharged within the fourth POD in 16.4% of cases, in 58.8% between the fifth and the eighth POD and after the eighth POD in 24.8% (Table 4).

**Table 4:** Postoperative recovery differences between groups

Variables	*n* = 1225	ICA (*n* = 862)	ECA (*n* = 363)	*p* value
Time to flatus passage (days)				< 0.0001
1	279 (22.8%)	239 (27.7%)	40 (11%)	
2–3	814 (66.4%)	554 (64.3%)	260 (71.6%)	
≥ 4	132 (10.8%)	69 (8%)	63 (17.4%)	
Time to bowel movement (days)				< 0.0001
1-2	263 (21.5%)	223 (25.9%)	40 (11%)	
3–5	851 (69.5%)	572 (66.3%)	279 (76.9%)	
> 5	111 (9.1%)	67 (7.8%)	44 (12.1%)	
Resumption of liquid diet (days)				< 0.0001
0	136 (11.1%)	128 (14.9%)	8 (2.2%)	
1–2	752 (61.4%)	577 (66.9%)	175 (48.2%)	
3–4	238 (19.4%)	113 (12.1%)	125 (34.4%)	
> 5	99 (8.1%)	44 (5.1%)	55 (15.2%)	
Resumption of solid diet (days)				< 0.0001
0	10 (0.8%)	9 (1%)	1 (0.3%)	
1–2	460 (37.6%)	396 (45.9%)	64 (17.6%)	
3–4	503 (41.1%)	332 (38.5%)	171 (47.1%)	
≥ 5	252 (20.6%)	125 (14.5%)	127 (35%)	
Hospital stay (days)				< 0.0001
0–4	201 (16.4%)	190 (22.1%)	11 (3%)	
5–8	720 (58.8%)	503 (58.3%)	217 (59.8%)	
≥ 9	304 (24.8%)	169 (19.6%)	135 (37.2%)	
Length of stay (days)				< 0.0001
(Median) [QR]	570 (46.5%)	6 [5–8]	8 [7–10]	
ERAS protocol				< 0.0001
Yes	655 (53.5%)	537 (62.3%)	118 (32.5%)	
No	570 (46.5%)	325 (37.7%)	245 (67.5%)	

### Postoperative complications

Postoperative complications have been recorded in 489 patients (39.9%); no complications have occurred in 736 patients (60.1%). Main complications were anastomotic bleeding (4%), anastomotic leakage (2.2%), bowel obstruction (1.7%), intra-abdominal abscess (1.8%), wound infection (4.3%) [28]. Minor complications included in grade 1 and 2 of Clavien–Dindo scale [29] were reported in 25.9% of all patients (Table 5).

**Table 5:** Postoperative complications

Complications	ECA (%)	ICA (%)	N (%)	*p* value
Wound infection	23 (6.3)	30 (3.5)	53 (4.3)	0.021
Bleeding	12 (3.3)	37 (4.3)	49 (4)	0.421
Leakage	6 (1.6)	21 (2.4)	27 (2.2)	0.394
Abdominal abscess	4 (1.1)	18 (2.1)	22 (1.8)	0.235
Bowel obstruction	5 (1.38)	16 (1.86)	21 (1.7)	0.556
Clavien–Dindo 1–2	134 (36.9)	183 (21.2)	317 (25.9)	< 0.0001
None	179 (49.3)	557 (64.6)	736 (60.1)	< 0.0001
Total	363 (100)	862 (100)	1225	

Twenty-nine patients (2%) were readmitted in hospital within 30 days, mainly for bowel obstruction and wound infection. Four required further surgery: 1 for anastomotic leakage and 3 for bowel obstruction (Table 6).

**Table 6:** Reasons for readmission within 30 days

30-days readmission causes	ECA (%)	ICA (%)	*N* (%)
Intestinal obstruction	1 (11.1)	9 (45)	10 (34.5)
Anemia and wound infection	5 (55.6)	3 (15)	8 (27.6)
Pneumonia	1 (11.1)	1 (5)	2 (6.9)
Anemia and rectal bleeding	1 (11.1)	1 (5)	2 (6.9)
Abdominal pain and fever	0 (0)	1 (5)	1 (3.4)
Nausea and diarrhea	1 (11.1)	2 (10)	3 (10.4)
Blood effusion	0 (0)	1 (5)	1 (3.4)
Intra-abdominal abscess	0 (0)	1 (5)	1 (3.4)
Anastomotic leakage	0 (0)	1 (5)	1 (3.4)
Total	9 (100)	20 (100)	29 (100)

Univariate analysis was performed for risk factors for postoperative complications on the sample stratified by clinical, surgical and personal characteristics. No statistically significant difference was found according to patient’s gender, age, BMI, co-morbidities, ASA score, previous abdominal surgery, benign or malignant disease and location. The likelihood of developing postoperative complications did not depend, in a statistically significant way, on the use of laparoscopic/robotic technique as well as the imaging technology and the type of energy source used. The use of laparoscopic/robotic technique as well as the imaging technology and the type of energy source used did not influence statistically the likelihood of developing postoperative complications (Table 7).

**Table 7:** Univariate and multivariate analysis for risk factors for postoperative complications

Patient characteristics	Reference category	Unadjusted OR			Adjusted OR		
		OR	95%	CI	OR	95%	CI
			Lower	Upper		Lower	Upper
Male	Female	1.0157	0.796	1.259	0.991	0.783	1.317
Age categories	< 65						
66–75		0.9308	0.689	1.256	0.965	0.707	1.317
> 76		0.9976	0.751	1.324	1.040	0.761	1.421
Comorbidity	None						
1		1.1666	0.879	1.547	1.1944	0.873	1.632
More than 1		0.8216	0.607	1.112	0.8368	0.582	1.202
ASA score	1						
2		0.7396	0.476	1.147	0.7446	0.472	1.173
3		0.7805	0.499	1.219	0.7911	0.475	1.315
4		0.7077	0.324	1.543	0.7781	0.335	1.803
Previous abdominal surgery	None						
Yes		0.8330	0.662	1.048	0.8470	0.669	1.072
Operative time (minutes)	90–180						
180–270		0.851	0.663	1.091	0.8455	0.658	1.093
> 270		0.744	0.453	1.224	0.7596	0.454	1.237
Blood losses (ml)	0–200						
201–299		1	0.166	6.007	1.209	0.198	7.375
≥ 300		0.937	0.561	1.566	0.9408	0.559	1.582
BMI	< 30						
≥ 30		1.1031	0.788	1.542			
Pathology	Benign						
Malignant		1.1549	0.816	1.632			
Location	Cecum						
Ascending colon		0.8936	0.689	1.157			
Trasversum		0.7481	0.551	1.014			
Technique used	Laparoscopic-videoassistited						
Robotic		0.8419	0.543	1.303			
Technology	FullHD						
3D		0.9684	0.742	1.263			
4K		0.6198	0.360	1.064			
Indocyanine Green	Yes						
No		1.1909	0.813	1.743			
Dissection technology	Radiofrequency						
Ultrasounds		1.174	0.927	1.486			
Monopolar/bipolar		1.064	0.618	1.830			
No. of lymph nodes harvested	< 12						
≥ 12		1.043	0.652	1.668			

The ileo-colic side-to-side anastomosis direction (anisoperistaltic or isoperistaltic), technique (stapled or hand-sewn) and the method of enterotomy closure did not influence the anastomotic bleeding and leakage (Table 8).

**Table 8:** Association between surgical variables and anastomotic complications

Variables	Bleeding			Leakage		
	Bleeding (*n* = 49)	No bleeding (*n* = 1176)	*p* value	Leakage (*n* = 27)	No leakage (*n* = 1198)	*p* value
Anastomosis						
Intracorporeal	37 (75.5%)	825 (70.2%)	0.421	21 (77.8%)	841 (70.2%)	0.394
Extra-corporeal	12 (24.5%)	351 (29.8%)		6 (22.2%)	357 (29.8%)	
Anastomosis direction						
Anisoperistaltic	4 (8.2%)	173 (14.7%)	0.202	6 (22.2%)	171 (14.3%)	0.264
Isoperistaltic	45 (91.8%)	1003 (85.3%)		21 (77.8%)	1027 (85.7%)	
Anastomosis confectioning						
Manual	3 (6.1%)	145 (12.3%)	0.191	3 (11.1%)	145 (12.1%)	0.876
Mechanical	46 (93.9%)	1031 (87.7%)		24 (88.9%)	1053 (87.9%)	
Enterotomy closure						
Manual	43 (87.8%)	1010 (85.9%)	0.712	25 (92.6%)	1028 (85.8%)	0.316
Mechanical	6 (12.2%)	166 (14.1%)		2 (7.4%)	170 (14.2%)	
Mesocolon closure						
No	28 (57.1%)	490 (41.7%)	0.082	10 (37%)	508 (42.4%)	0.287
Yes, continuous	11 (22.5%)	412 (35%)		13 (48.2%)	410 (34.2%)	
Yes, interrupted	10 (20.4%)	274 (23.3%)		4 (14.8%)	280 (23.4%)	
Enterotomy manual closure^a^						
Single layer	8 (19%)	202 (19.5%)	0.935	6 (24%)	204 (19.4%)	0.569
Double layer	34 (81%)	831 (80.5%)		19 (76%)	846 (80.6%)	
Enterotomy hand-sewn suture^b^						
Continuous	39 (95.1%)	906 (90.8%)	0.342	21 (91.3%)	924 (90.9%)	0.953
Interrupted	2 (4.8%)	92 (9.2%)		2 (8.7%)	92 (9.1%)	

^a^9 missing values

^b^12 missing values

Adjusted logistic regression analysis showed that age, co-morbidities, previous abdominal surgery, gender, ASA score, operative time and blood loss did not influenced the likelihood of developing postoperative complications (Table 7).

To analyse the length of intervention in relation to the surgical technique, the sample was purified from conversions and intra-operative complications which would influence the results by increasing the operating time. The intervention lasted 90–180 min in 60.2% of ICA and 68.1% of ECA, it lasted 181–270 min in 33.3% of ICA and 27.9% of ECA, more than 270 min in 6.5% of ICA and 4% of ECA. Operating time in patients receiving an ICA resulted significant longer than in patients receiving an ECA (*p* = 0.037) (Table 9).

**Table 9:** Differences in length of intervention between groups

Variables	Anastomosis		*p* value
	ICA(846)^a^	ECA(298)^a^	
Lenght of intervention			0.037
90–180 min	509 (60.2%)	203 (68.1%)	
181–270 min	282 (33.3%)	83 (27.9%)	
> 270 min	55 (6.5%)	12 (4%)	

^a^Sample purified from conversions and intra-operative complications

Significant difference was found between ICA and ECA groups about postoperative complications. ICA group showed a lower rate of total complications and of Clavien–Dindo grade I–II, whereas higher rate of wound infections was associated to ECA technique. No differences were found among bleeding, leakage, abdominal abscess and bowel obstruction (Table 5).

Statistically significant differences have been found comparing ICA and ECA groups in relation to postoperative outcome. Patients in ICA group showed a shorter time of bowel function recovery, of resumption of liquid and solid diet, and of median length of stay (6 vs. 8, *p* < 0.0001) (Table 4).

Considering that ICA group had fewer complications than ECA group, we analyzed the postoperative outcome excluding patients converted to laparotomy, who had intra-operative and postoperative complications, and who were admitted into the intensive care unit in order to rule out potential confounding factors. Even in this sample, ICA is significantly associated with a better short-term outcome (*p* < 0.0001) (Table 10).

**Table 10:** Postoperative recovery differences between groups

Variables	ICA (*n* = 486)^a^	ECA (*n* = 130)^a^	*p* value
Time to flatus passage (days)			< 0.0001
1	156 (32.1%)	16 (12.3%)	
2–3	311 (64%)	101 (77.7%)	
≥ 4	19 (3.9%)	13 (10%)	
Time to bowel movement (days)			0.013
1–2	151 (31.1%)	24 (18.5%)	
3–5	319 (65.6%)	102 (78.5%)	
> 5	16 (3.3%)	4 (3%)	
Resumption of liquid diet (days)			< 0.0001
0	81 (16.7%)	1 (0.7%)	
1–2	352 (72.4%)	81 (62.3%)	
3–4	38 (7.8%)	46 (35.4%)	
> 5	15 (3.1%)	2 (1.6%)	
Resumption of solid diet (days)			< 0.0001
0	8 (1.6%)	0 (0%)	
1–2	251 (51.6%)	29 (22.3%)	
3–4	190 (39.1%)	87 (66.9%)	
≥ 5	37 (7.6%)	14 (10.8%)	
Length of stay (days)			< 0.0001
0–4	152 (31.3%)	6 (4.6%)	
5–8	295 (60.7%)	87 (66.9%)	
≥ 9	39 (8%)	37 (28.5%)	
Length of stay (days)			
(median) [QR]	5 [4 7]	7 [6 9]	< 0.0001

^a^sample purified from conversions, intra and postoperative complications, admission in intensive care unit

Because of the great difference among the percentage of the ICA vs ECA cohorts receiving ERAS, a separated analysis of postoperative outcomes in the two groups ERAS and no-ERAS was carried out. In the group following ERAS protocol, all variables regarding postoperative outcome were significantly better in patients chosen for ICA. Analyzing the no-ERAS cohorts, patients chosen for ICA had faster resumption of liquid and solid diet, with shorter hospital stay even if the median LOS was equal to ECA group. No statistical significance had been found regarding the resumption of bowel movement which, anyway, resulted earlier in ICA group (Table 11).

**Table 11:** Postoperative recovery in ERAS and no-ERAS groups in relation to anastomotic technique

Variables	ERAS (655)			no-ERAS (570)		
	ICA (537)	ECA (118)	*p* value	ICA (325)	ECA (245)	*p* value
Time to flatus passage (days)			< 0.001			0.490
1	208 (38.7%)	13 (11%)		31 (9.5%)	27 (11%)	
2–3	310 (57.7%)	87 (73.7%)		244 (75.1%)	173 (70.6%)	
> 4	19 (3.6%)	18 (15.3%)		50 (15.4%)	45 (18.4%)	
Time to bowel movement (days)			< 0.001			0.110
1–2	170 (31.7%)	15 (12.7%)		53 (16.3%)	25 (10.2%)	
3–5	345 (64.2%)	95 (80.5%)		227 (69.8%)	184 (75.1%)	
> 5	22 (4.1%)	8 (6.8%)		45 (13.9%)	36 (14.7%)	
Resumption of liquid diet (days)			< 0.001			< 0.001
0	128 (23.8%)	7 (5.9%)		0 (0%)	1 (0.4%)	
1–2	379 (70.6%)	71 (60.2%)		198 (60.9%)	104 (42.4%)	
3–4	23 (4.3%)	31 (26.3%)		90 (27.7%)	94 (38.4%)	
> 5	7 (1.3%)	9 (7.6%)		37 (11.4%)	46 (18.8%)	
Resumption of solid diet (days)			< 0.001			< 0.001
0	9 (1.7%)	0 (0%)		0 (0%)	1 (0.4%)	
1–2	340 (63.3%)	46 (39%)		56 (17.2%)	18 (7.3%)	
3–4	158 (29.4%)	44 (37.3%)		174 (53.5%)	127 (51.8%)	
> 5	30 (5.6%)	28 (23.7%)		95 (29.3%)	99 (40.5%)	
Hospital stay (days)			< 0.001			0.040
0–4	179 (33.3%)	9 (7.6%)		11 (3.4%)	2 (0.8%)	
5–8	306 (57%)	80 (67.8%)		197 (60.6%)	137 (55.9%)	
> 9	52 (9.7%)	29 (24.6%)		117 (36%)	106 (43.3%)	
Leight of stay (days)			< 0.001			0.0163
(median) [QR]	5 [4–7]	7 [6–8]		8 [6–10]	8 [7–10]	

**Fig. 1:**
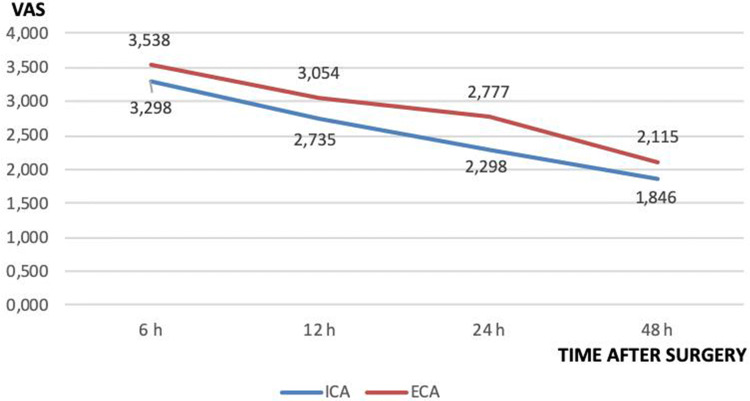
Mean pain severity divided by group within 48 h post-surgery

Postoperative pain within 48 h after surgery was similar in the two groups (Fig. 1), but the subgroup analysis of those patients in whom opioids were not prescribed demonstrated a significant reduction of the mean reported pain in the ICA group except for the detection of pain within 6 h (Table 12).

**Table 12:** Distribution of VAS scale (mean SD) VAS by intervals post-surgery

Opiod administration	Yes		*p* value	No		*p* value
Time classes (h)	Type of anastomosis			Type of anastomosis		
	ICA (*n* = 213)	ECA (*n* 74)		ICA (*n* = 273)	ECA (*n* 56)	
6	3.357 (1.921)	3.445 (2.088)	0.7371	3.253 (1.734)	3.661 (1.947)	0.1174
12	2.943 (1.701)	2.919 (1.907)	0.9169	2.571 (1.402)	3.232 (1.737)	0.0023
24	2.455 (1.579)	2.689 (1.719)	0.2847	2.176 (1.311)	2.893 (1.626)	0.0004
48	2.098 (1.468)	1.851 (1.201)	0.1922	1.648 (0.936)	2.464 (1.584)	< 0.0001

## Discussion

Nowadays, minimally invasive surgery is accepted as standard approach for benign and malignant colon diseases on the basis of proven short and long-term outcomes.

In 2003 the totally laparoscopic side-to-side ileo-colic stapled anastomosis was proposed after elective LRH [6] and, today, it is still considered a challenging procedure which requires proper learning curve. In facts, with ICA the rate of intraperitoneal contamination is higher, especially in patients without mechanical bowel preparation before surgery [10, 21–23].

ICA was indicated first in case of obesity, to avoid the bigger incision necessary to extract the ileo-colic specimen and the need of mesentery traction during ECA confectioning. Furthermore, it was reported that ECA was associated with a higher rate of wound infection particularly in obese patients [7–9, 13, 24, 30].

Over the years, retrospective monocenter studies and a few meta-analysis comparing ICA vs ECA, showed best short terms outcomes in patients undergoing ICA. Just a single study showed a lower rate of anastomotic leakage in case of ICA [11]. A recent meta-analysis confirms a lower perioperative morbidity in ICA group [20]. Nonetheless, no prospective multi-center observational trial has compared totally intracorporeal vs extra-corporeal side-to-side ileo-colic anastomosis technique to define advantages and disadvantages and to offer recommendations to surgeons and stakeholders [23, 24, 31, 32].

In order to support the clinical practice of the members and to guarantee the best patients care, the Scientific Committee of SICE promoted the present observational prospective cohort study to collect data and frame the current surgical practice in Italy. 85 accredited surgical units with at least two active SICE members in each center were involved [33].

During the observational period, the participating surgeons (210 accredited laparoscopic surgeons) recruited a large cohort of 1225 consecutive cases respecting the inclusion criteria. According to our knowledge, the present observational prospective study has the largest sample of elective right hemicolectomy performed with mini-invasive technique that compares ICA versus ECA.

The present study involved high volume colorectal units with expertise in colorectal and advanced laparoscopic surgery, therefore surgeons were probably predisposed to perform more complex surgical procedures, like ICA during LRH. This represents a possible bias for the following results.

The results of the survey demonstrated that the large majority of the Italian surgeons involved performs a side-to-side isoperistaltic totally intracorporeal stapled anastomosis after any indications for elective laparoscopic right hemicolectomy with hand-sewn closure of the enterotomies (preferred double layer with continuous suture). The majority of the surgeons used Full HD vision technology; only in the 7.5% of the cases a robotic setting was used.

The length of intervention resulted higher in ICA group, consistent with the longer time necessary to perform the anastomosis with the completely intracorporeal technique.

The analysis of the characteristics of patients stratified according to the anastomotic technique (ICA vs. ECA) allowed to select two homogeneous groups for the statistical analysis of perioperative complications.

Our sample comprised a large portion of elderly patients (> 65 years old) with co-morbidities and previous abdominal surgery, but none of those parameters influenced the postoperative complications rate in subgroups multivariate analysis in relation to the anastomotic technique (ICA vs. ECA). The multivariate analysis did not identify any predictive factor for anastomosis complications. This finding is in contrast with previous reports [34–36] and could be related to a possible bias of this study involving high volume colorectal units with expertise in colorectal surgery.

We observed that the total number of complications as well as minor complications (Clavien–Dindo grade I–II) were influenced by the anastomotic technique, with a lower rate in ICA group. On the contrary, wound infection resulted higher in ECA group and it also represented the most frequent complication. For other complications like, in order of frequency, anastomotic bleeding and leakage, no statistical difference had been found between the two groups.

Hospital readmission rate within 30 days was low (2%).

In accordance to what reported in literature [4, 7, 24, 31], we observed better short term outcomes and a significant decrease of the hospital stay in ICA group than ECA, both in the total sample and, as further confirmation, in a sample purified from any complications.

ERAS protocol could be a confounding factor for these results because, even if the population looks homogeneous with 53.3% following ERAS protocol and 46.5% not, actually it is more applied in ICA group than ECA (62.3% vs. 32.5%). Nevethless, further analysis confirmed that ICA positively affects the postoperative outcome independently from ERAS protocol application. Anyway, a minimally invasive approach, irrespective of the anastomotic technique, is recommended to improve the clinical outcome after ERAS application [37].

Lastly, data showed a statistically significant improvement in the ICA group for the postoperative pain control from the 12th h after surgery with maximum benefits after the 48th in patients not treated with opioids.

## Conclusion

The present Italian multicentric prospective observational study frames the laparoscopic surgeons’ attitude about the technical aspects of ileo-colic anastomosis after elective laparoscopic right hemicolectomy for any indications. The skill of the surgeons has allowed the standardization of the totally intracorporeal technique in each high volume laparoscopic center: a side-to-side isoperistaltic stapled ICA with hand-sewn enterotomy closure is the most frequently adopted technique.

No predictive factors for anastomotic complications have been identified among the population characteristics.

The study confirms that intracorporeal anastomosis has advantages in relation to the onset of postoperative complications, especially Clavien–Dindo I–II. The technique by itself has positive influence on the postoperative recovery: it showed better short term outcome with significant reduction of the length of hospital stay, independently from ERAS protocol application.

Finally, these results allow some speculation: the diffusion of the ICA technique is based on better perioperative outcomes and, even more, may be parallel with an increased confidence with advanced laparoscopic colorectal surgery enhanced by vision technologies.
